# Context Dependent Role of the CD36 - Thrombospondin - Histidine-Rich Glycoprotein Axis in Tumor Angiogenesis and Growth

**DOI:** 10.1371/journal.pone.0040033

**Published:** 2012-07-10

**Authors:** James Scott Hale, Meizhang Li, Maksim Sinyuk, Willi Jahnen-Dechent, Justin Durla Lathia, Roy Lee Silverstein

**Affiliations:** 1 Department of Biological, Geological and Environmental Sciences, Cleveland State University, Cleveland, Ohio, United States of America; 2 Department of Cell Biology, Lerner Research Institute, Cleveland Clinic Foundation and Department of Molecular Medicine, Cleveland Clinic Lerner College of Medicine, Cleveland, Ohio, United States of America; 3 Helmholtz Institute for Biomedical Engineering, Rheinisch-Westfälische Technische Hochschule Aachen University Hospital, Aachen, Germany; 4 Department of Medicine, Medical College of Wisconsin, Milwaukee, Wisconsin, United States of America; Ottawa Hospital Research Institute, Canada

## Abstract

The angiogenic switch is a promising therapeutic target in cancer. Work by our laboratory and others has described an important endogenous anti-angiogenic pathway mediated by interactions of CD36, a receptor on microvascular endothelial cells, with proteins containing thrombospondin (TSP) type I repeat domains (TSR). Recent studies revealed that circulating Histidine Rich Glycoprotein (HRG) inhibits the anti-angiogenic potential of the CD36-TSR pathway by functioning as a decoy receptor that binds and sequesters TSR proteins. As tumors of different origin display variable expression profiles of numerous targets, we hypothesized that the TSP-CD36-HRG axis regulates vascularization and growth in the tumor microenvironment in a context, or tumor type, dependent manner. Growth of Lewis Lung Carcinoma (LL2) and B16F1 Melanoma tumor cell implants in syngeneic wild type (WT), *hrg*, or *cd36* null mice were used as a model to interrogate this signaling axis. LL2 tumor volumes were greater in *cd36* null mice and smaller in *hrg* null mice compared to WT. Immunofluorescent staining showed increased vascularity in *cd36* null vs. WT and WT vs. *hrg* null mice. No differences in tumor growth or vascularity were observed with B16F1 implants, consistent with lack of expression of TSP-1 in B16F1 cells. When TSR expression was induced in B16F1 cells by cDNA transfection, tumor growth and vascularity were similar to that seen with LL2 cells. These data show a role for CD36-mediated anti-angiogenic activity in the tumor microenvironment when TSR proteins are available and demonstrate that HRG modulates this activity. Further, they suggest a mechanism by which tumor microenvironments may regulate sensitivity to TSR containing proteins.

## Introduction

Angiogenesis is the physiologic process by which new vessels sprout from the existing vasculature. In the normal adult setting, the vasculature is maintained in a quiescent state through a balance of angiogenic inhibitors, such as thrombospondin (TSP)-1, and inducers, such as vascular endothelial growth factor (VEGF). This balance between pro and anti- angiogenic stimuli is important in processes such as pregnancy and wound healing. Loss of homeostatic balance resulting in excessive or insufficient angiogenesis has been implicated in numerous diseased states such as ulcerative colitis, diabetic retinopathy, obesity, psoriasis, rheumatoid arthritis, stroke, coronary artery disease and cancer [1].

It is well established that solid tumors will grow to 1–2 mm by simple diffusion but require a blood supply in order to expand further and metastasize [2]. To this end tumors express pro-angiogenic substances such as basic fibroblast growth factor (bFGF) and VEGF which recruit blood vessels to the lesion through the induction of microvascular endothelial cell proliferation, migration and tube formation [3]. Previous studies have shown ablation of pro-angiogenic phenotypes by endothelial cell membrane receptor CD36 [4], [5]. CD36, an 88 kDa class B scavenger receptor, is expressed on numerous vascular cell types including macrophages, platelets and microvascular endothelial cells. CD36 recognizes at least three classes of extracellular ligands – oxidized phospholipids, long chain fatty acids and proteins containing the so-called thrombospondin type I repeat (TSR) [6]–[10]. These receptor-ligand interactions mediate effects in a cell type specific manner. With regard to microvascular endothelial cells, a specific region of CD36 known as the CLESH domain interacts with high affinity with TSR domains of at least three endogenous anti-angiogenic proteins - thrombospondins-1 and -2 and vasculostatin [8]–[10]. These interactions initiate a complex intracellular signaling cascade involving the Src family tyrosine kinase P59^fyn^ and p38 mitogen-activated protein kinase (MAPK) resulting in direct activation of caspase 3 protease leading to induction of apoptosis [11]. CD36 mediated anti-angiogenic activity also involves induction of pro-apoptotic receptors, including TNFR-1 and Fas [12], [13]. These pro-apoptotic signals interrupt angiogenic responses induced by pro-angiogenic growth factors, such as bFGF and VEGF.

**Figure 1:**
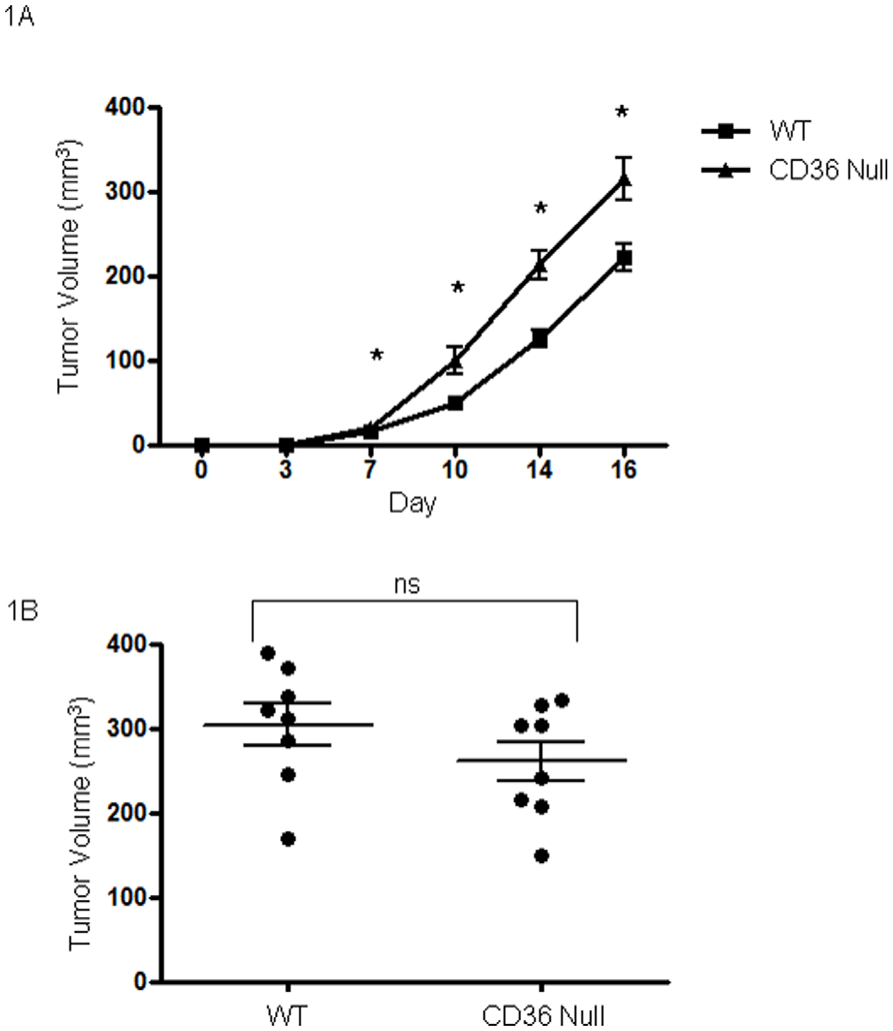
*Cd36* deletion in mice enhances syngeneic tumor growth. Lewis Lung carcinoma cells (**A**) or B16F1 melanoma cells (**B**) were injected in the backs of *cd36* null or wild type C57BL/6 mice (50,000 cells/mouse). Tumor volumes were assessed over 17 days following implantation. *P<0.05.

**Figure 2:**
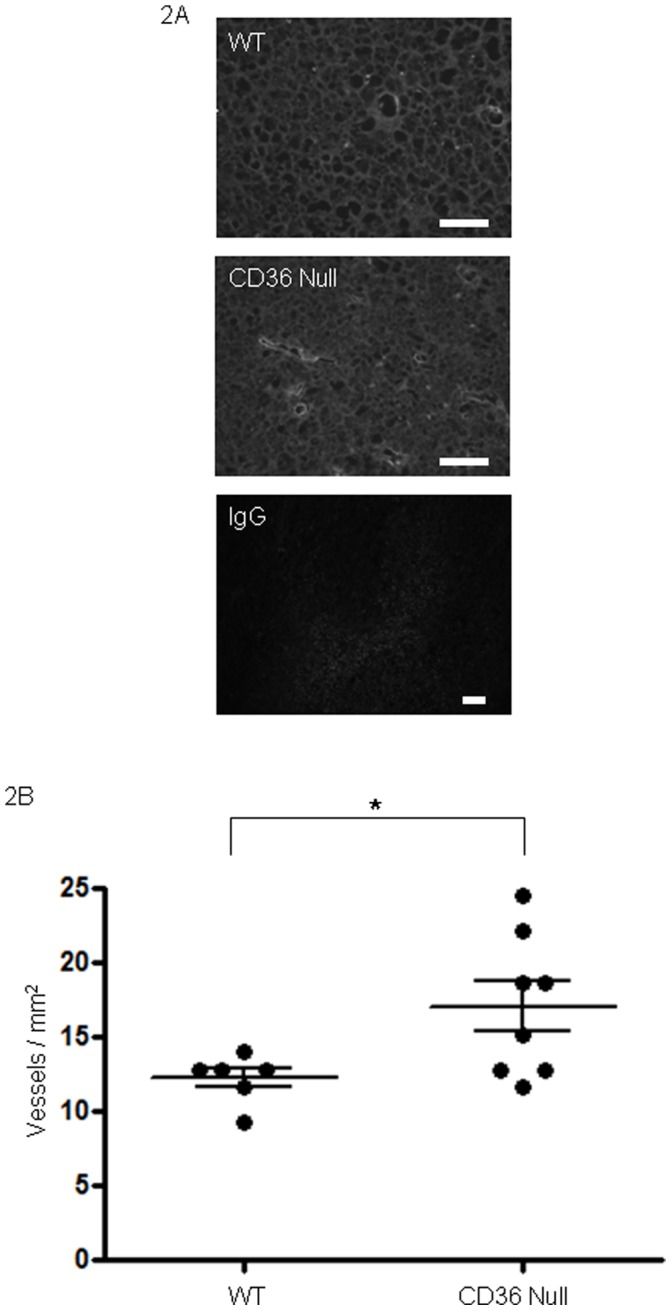
*Cd36* deletion in mice enhances Lewis Lung tumor vascularity. (A) Lewis Lung tumors as in Figures 1 were dissected, sectioned and examined by immunofluorescence microscopy using anti-VEGF receptor antibody (green) to detect blood vessels. DAPI stained nuclei are blue. Magnification bars represent 100 µm. IgG control is shown in bottom panel as negative control. (**B**) Vessel densities measured as vessels per mm^2^. Median vessel density: wt 12.83, cd36 null 16.91.

Despite abundant evidence in mouse models and human tumors that down-regulation of TSR-protein expression by genetic or epigenetic pathways in cancer cells promotes angiogenesis and thereby promotes tumor growth and metastasis, little is known whether modulating TSR interactions with its receptor, CD36, can influence tumor behavior [14]–[17]. In data described in this manuscript we tested the hypothesis that genetic deletion of *cd36* or of *hrg*, a gene encoding a circulating CD36 decoy protein, would modulate tumor angiogenesis and tumor growth in syngeneic mouse tumor implantation models in a context dependent manner based on differential expression of TSR proteins by tumor types.

Histidine-Rich Glycoprotein (HRG) is a 75 kDa protein synthesized by hepatocytes that circulates in plasma at relatively high concentrations (100–200 µg/ml) [18]. There are also abundant stores of HRG in the alpha granules of platelets (∼371 ng/10^9^ platelets) that can be released into specific microenvironments in response to platelet activation [19], [20]. HRG is a modular protein that binds to proteoglycans, matrix proteins, divalent cations, and coagulation proteins. It possesses a domain analogous to the CLESH domain of CD36 that is able to bind TSRs of thrombospondin-1 and 2 and vasculostatin [8]–[10]. It is through this domain that HRG acts as a soluble decoy receptor for TSR domains, thereby blocking their binding to CD36 and regulating anti-angiogenic signaling on microvascular cells. As such, we hypothesized that tumors formed in mice lacking HRG will display increased CD36-TSP signaling resulting in decreased in vascularization and tumor growth.

In the present manuscript we show that genetic deletion of *cd36* or *hrgp* in C57BL/6 mice effected tumor growth and vascularity. As predicted by our model tumor growth was increased in *cd36* null mice and decreased in *hrg* null mice. Additionally, we demonstrated that these effects depended on tumor cell secretion of TSR-containing protein.

**Figure 3:**
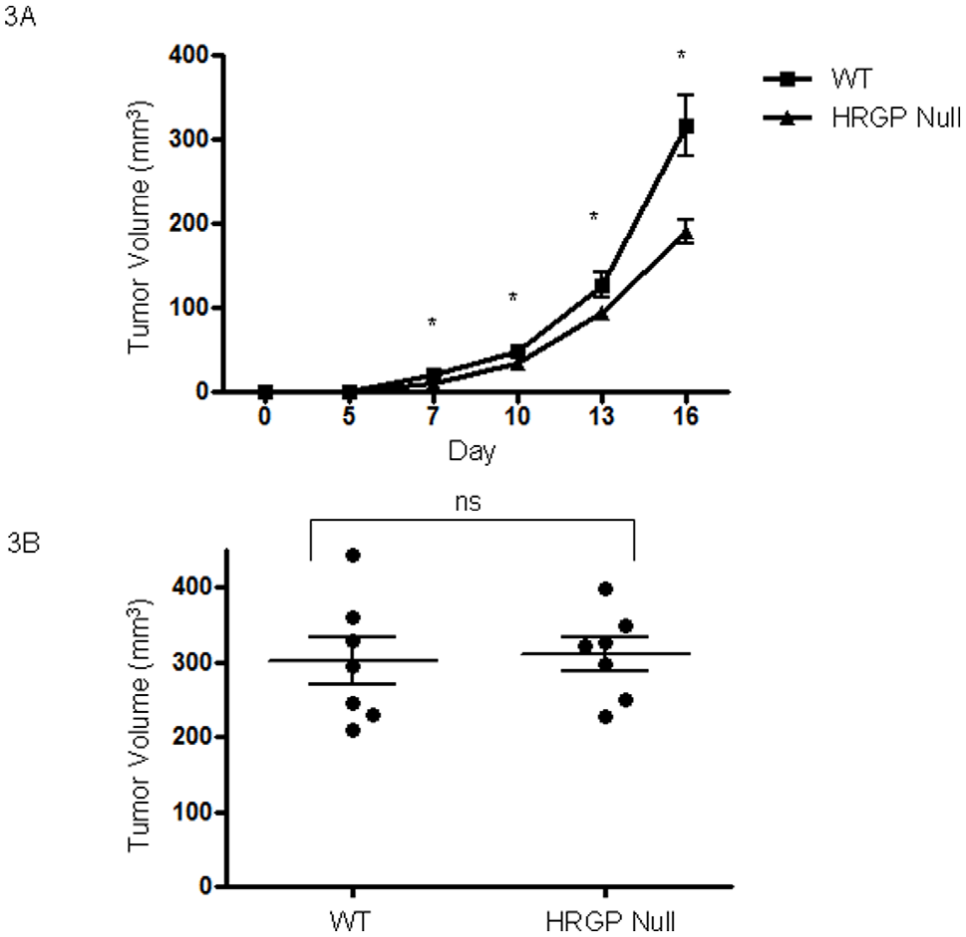
*Hrg* deletion in mice suppresses syngeneic tumor growth. Lewis Lung carcinoma cells (**A**) or B16F1 melanoma cells (**B**) were injected in the backs of *hrg* null or wild type C57BL/6 mice (50,000 cells/mouse). Tumor volumes were assessed over 16 days following implantation. *P<0.05.

**Figure 4:**
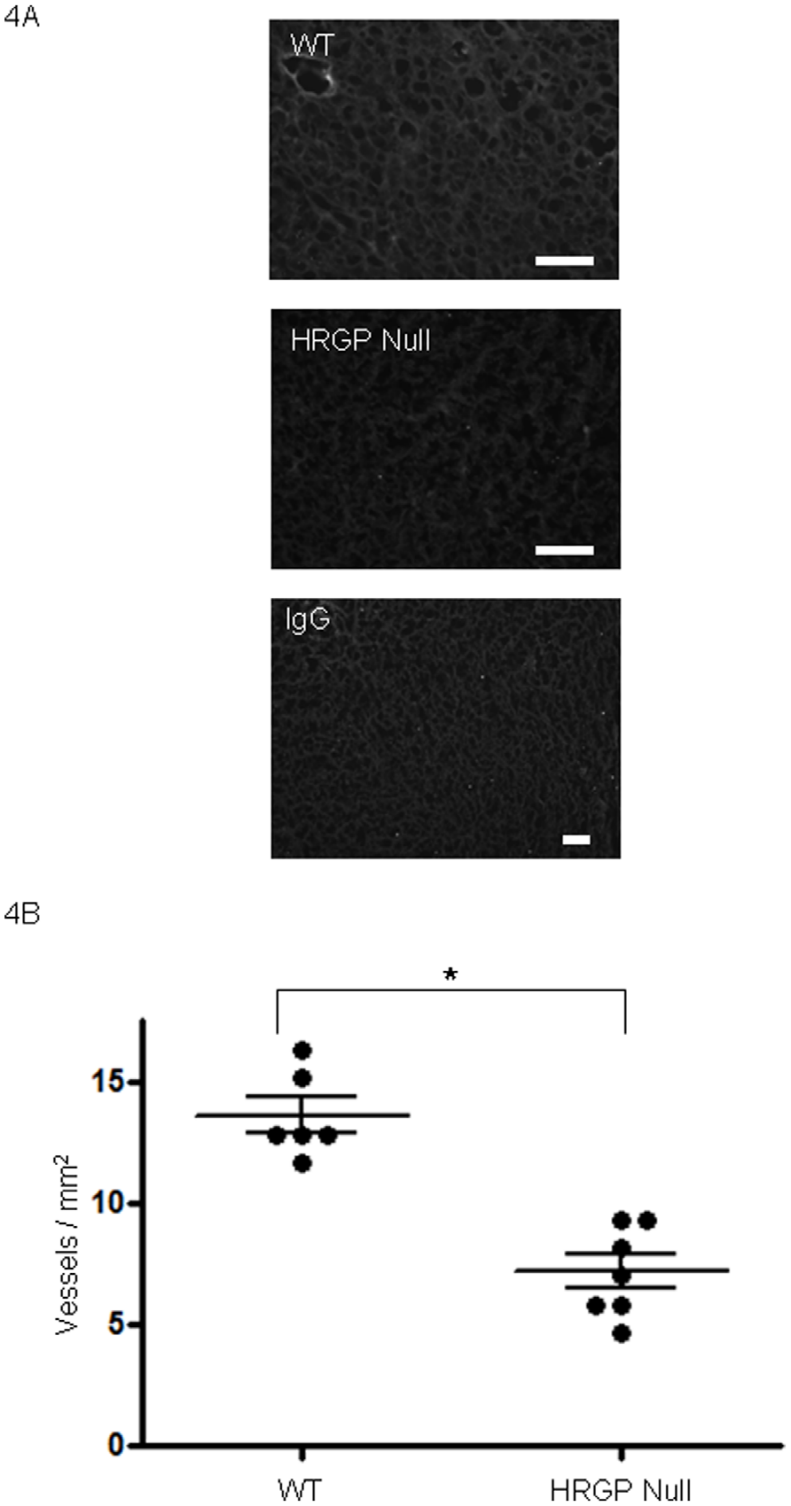
*Hrg* deletion in mice suppresses Lewis Lung tumor vascularity. (**A**) Lewis Lung tumors as in Figure 3 were dissected, sectioned and examined by immunofluorescence microscopy using anti-VEGF receptor antibody (green) to detect blood vessels. DAPI stained nuclei are blue. Magnification bars represent 100 µm. IgG control is shown in bottom panel as negative control. (**B**) Vessel densities measured as vessels per mm2. Median vessel density: wt 12.33, cd36 null 6.99.

## Methods

### Materials

Mouse anti-VEGF receptor 2 antibody was from Cell Signaling Technology. Rabbit anti- VE-Cadherin and TSP polyclonal antibodies were from Abcam. Goat anti-rabbit IgG Alexafluor 488 conjugate and DAPI Prolong Anti-fade mounting media were from Invitrogen. Goat anti-rabbit horseradish peroxidase (HRP) was from Promega. Tissue Tek Optimal cutting temperature compound (OCT) was from Fisher Scientific. Heparin, sucrose and paraformaldehyde were from Sigma.

**Figure 5:**
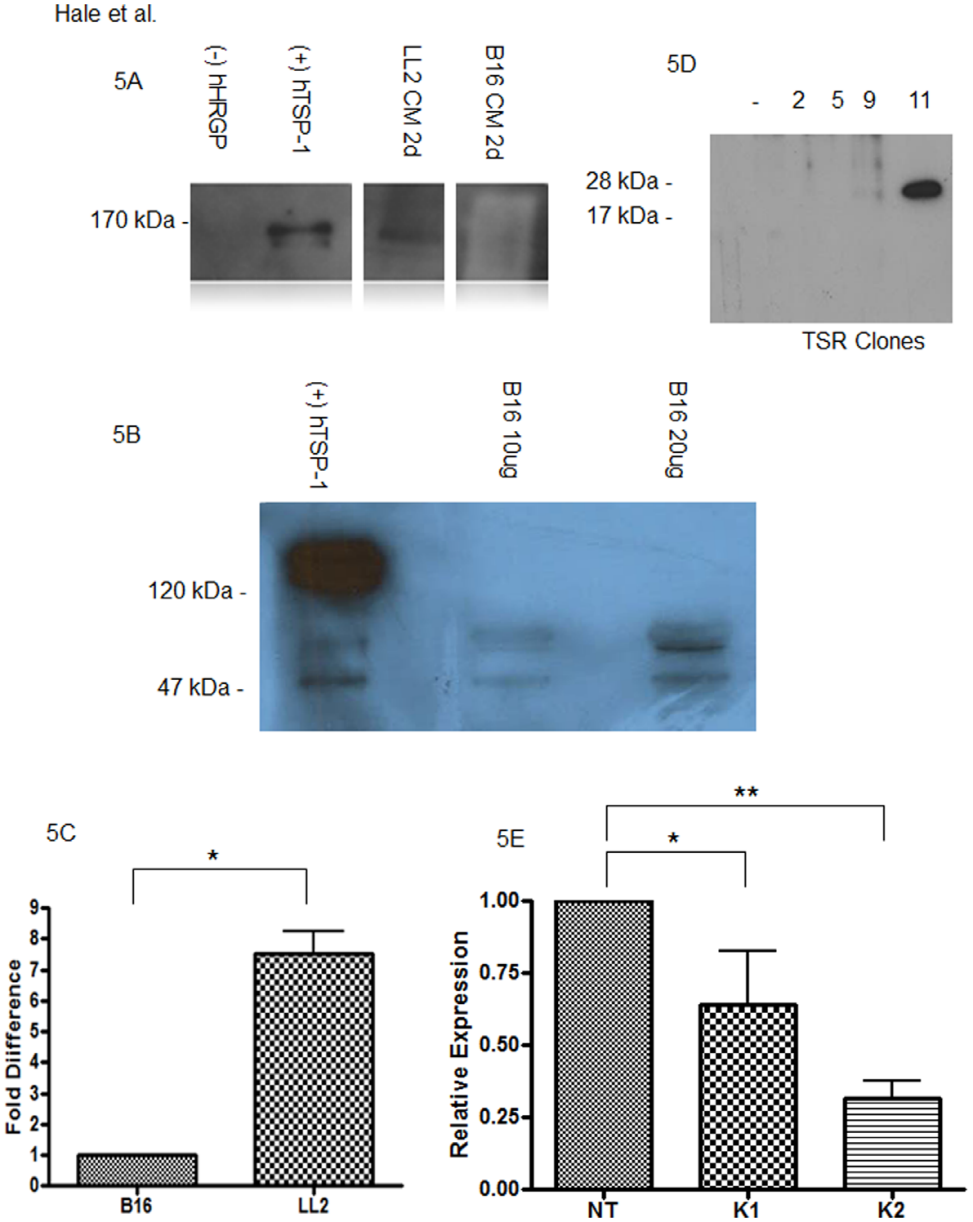
Thrombospondin-1 expression in LL2 and B16F1 melanoma cells and tumors. (**A**) Lewis Lung (LL2) or B16F melanoma cells were cultured in serum free media for 24 hours (1d) at which point proteins in post culture media (CM) were precipitated by TCA, separated under reducing conditions by SDS/PAGE and analyzed by immunoblot using anti-TSP-1 antibody. TSP-1 monomers were detected at 170 kDa in the media conditioned by LL2 cells, but not B16F1 cells. Purified human HRG and TSP were used as controls. (**B**) B16F1 melanoma tumor tissue was analyzed by western blot analysis for TSP expression. Intact TSP was not observed at 150 kDa, however possible degredation products were observed around 55 kDa. (**C**) B16F1 and LL2 tumor tissue was analyzed by RT-PCR for expression of TSP. TSP was detected in both tumor types, approximately 7 fold higher in LL2. (**D**) Conditioned media was collected from 4 different antibiotic resistant clones of TSR transfected B16F melanoma cells and analyzed by immunoblot as in panel A. Clone 11 expressed abundant anti-TSP reactive material at the appropriate molecular weight of recombinant TSR and was utilized for subsequent tumor studies. (**E**) TSP knockdown efficiency was analyzed by RT-PCR with statistical significance as indicated; **P<0.05; *P = 0.06. In both instances of TSP knockdown 1 (K1 and K2), reductions in TSP message levels were detected as compared with nontargeted control (NT) cells.

### Tumor Cells

Lewis Lung Carcinoma cells (LL/2) (CRL-1642) and B16F1 melanoma cells (CRL-6323) were obtained from the ATCC and maintained in Dulbecco’s Modified Eagle Medium (Gibco) supplanted with 10% fetal bovine serum (Atlanta biologicals) and 0.5% penicillin/streptomycin (10,000 U/ml, Gibco). Cells were incubated at 37°C, 95% humidity and 5% CO2, grown in 75 cm2 cell culture flasks (Corning) and passaged twice weekly. Cultures past 15 passages were not utilized. Stably transfected TSR-expressing B16F1 melanoma cell lines were generated by transfecting the cells with pSecTag2 secretory plasmid (Invitrogen) into which a cDNA encoding the TSR domains of mouse TSP-1 (amino acids 375–551) was cloned. Primers used for the cloning were ATATTGAAGCTTGCCCAGCGACTCTGCTGAC and ATATTGCTCGAGGTCCATCAATTGGGCAGTC. Transfection was done using the Fugene 6 reagent (Promega) as per manufacturer’s directions. Transfected clones were selected by antibiotic resistance using Zeocin (Invitrogen) at a concentration of 600 µg/ml. TSR expressing clones were identified by reverse transcription polymerase chain reaction and confirmed by western analysis of serum free cultured media.

**Figure 6:**
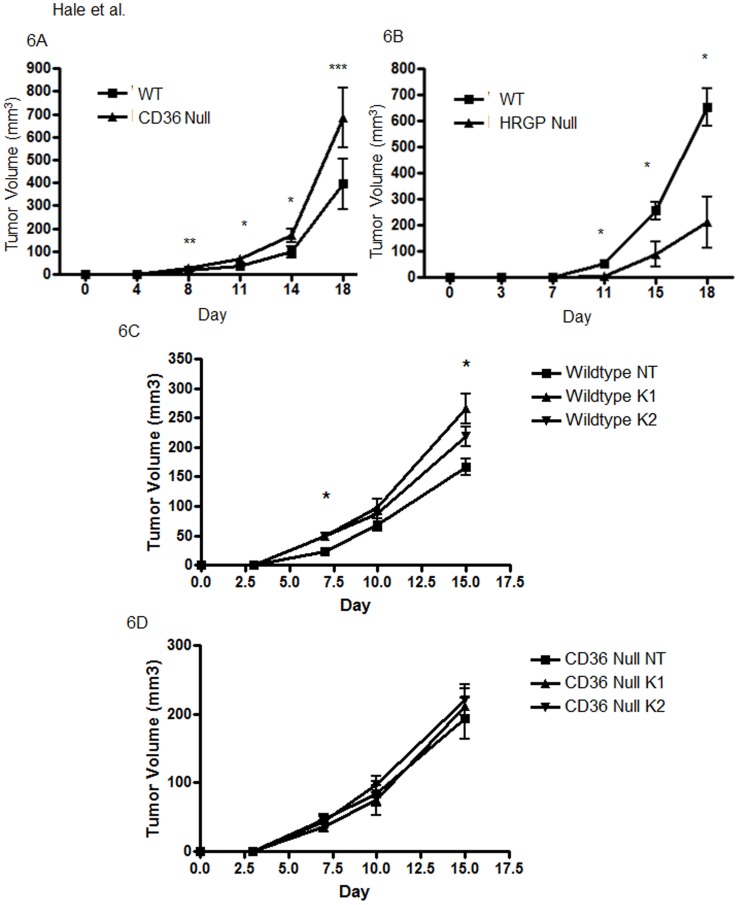
TSR transfected B16F1 melanoma cells show enhanced tumor growth in *cd36* null mice and suppressed tumor growth in *hrgp* null mice. 50,000 cells from a stably transfected B16f1 melanoma cell line (Clone 11) were injected in the backs of *cd36* null (A) or *hrgp* null (B) mice. C57BL/6 mice were used as controls. Tumor volumes were assessed at timed points as in Figures 1 and 3. *P<0.05; **P = 0.08; ***P = 0.06. LL2 cells stabily transfected with nontargeted (NT) or TSP targeted constructs, K1 and K2, constructs were similarly injected subcutaneously onto the backs of wildtype (WT) and cd36 null mice (Null). (**C**) WT-NT tumors grew smaller than WT-K1 and WT-K2 tumors, with statistically significant differences seen at days 7 and 15 for both NT vs K1 and NT vs K2. (**D**) No differences were observed between NT, K1 and K1 in cd36 null mice.

**Figure 7:**
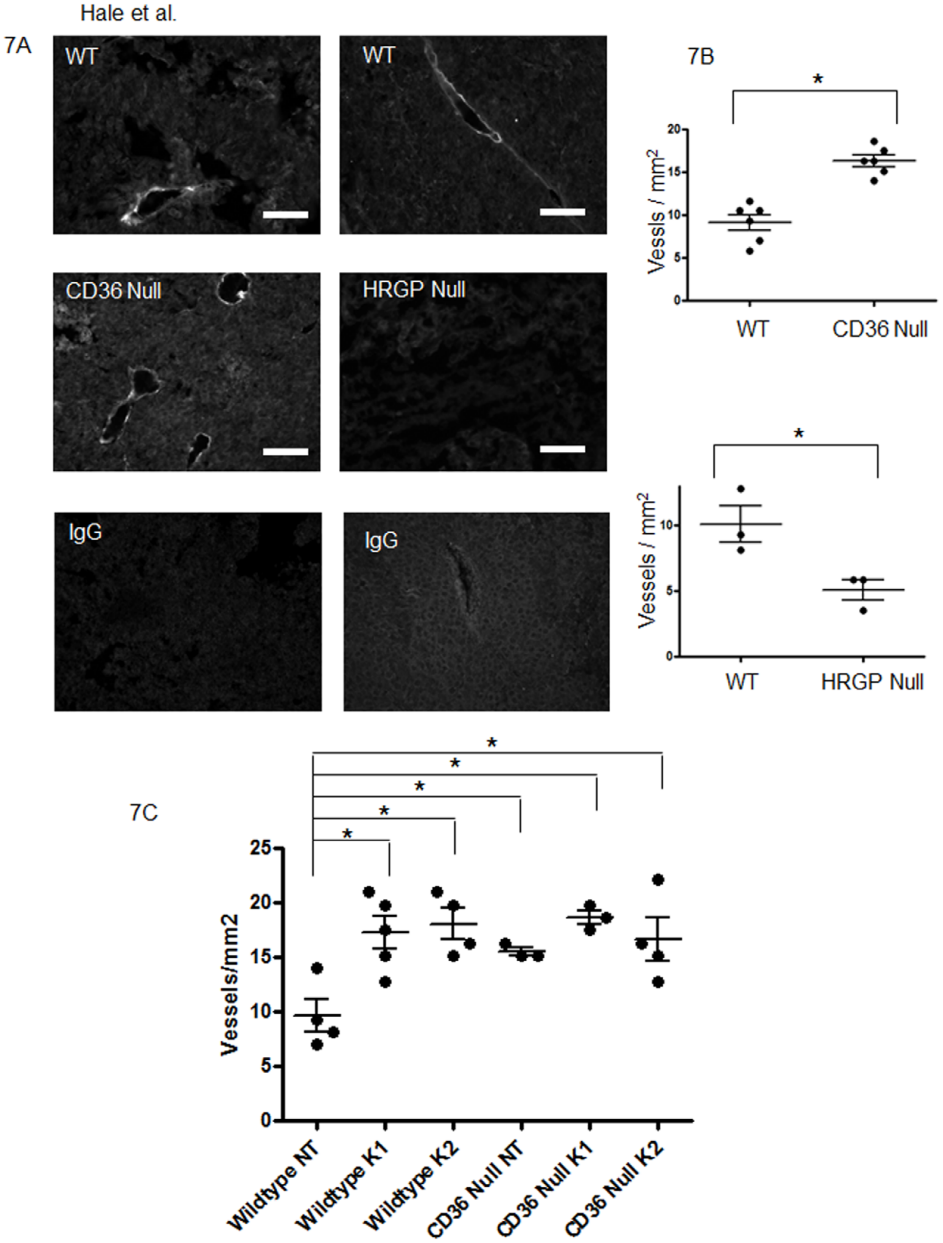
TSR transfected B16F1 melanoma cells show enhanced tumor vascularity in *cd36* null mice and suppressed tumor vascularity in *hrg* null mice. (**A**) Tumors from TSR transfected B16F1 melanoma cells as in Figure 6 were dissected, sectioned and examined by immunofluorescence microscopy using anti-VE-Cadherin antibody (green) to detect blood vessels. DAPI stained nuclei are blue. Magnification bars represent 100 µm. IgG control is shown in bottom panels as negative control. (**B**). Median vessel density: wt 8.99, cd36 null 16.32. Median vessel density: wt 9.33, hrgp null 5.83. (**C**) Tumors from lentiviral (NT, K1 and K2) transfected LL2 cells from wildtype and cd36 null mice were examined using anti-VE-Cadherin antibody to detect blood vessels. Vessel densities measured as vessels per mm^2^.

### Animals

All experiments and handling of mice were approved by the Institutional Animal Care and Use Committee (IACUC) of Cleveland Clinic, protocol 2009-0060. Mice were housed in a facility fully accredited by AALAC and in accordance with all federal and local regulations. All mouse strains used were of the same genetic background as the tumor cells - C57BL/6. Generation of *cd36* null and *hrg* null mice has previously been described [21], [22]. Mice null for *hrg* were initially of the 129/B6 background and were backcrossed 10 generations onto C57BL/6 background.

### TSP-1 and TSR Expression Analysis

Secretion of TSP-1 or recombinant TSR peptide by mouse tumor cells was assessed after culture in serum free media for 48 hours. Post culture media was collected and proteins precipitated with trichloroacetic acid. Precipitated samples were washed twice with acetone, resuspended in laemmli sample buffer and then electrophoresed on SDS-PAGE (10%) gels under reducing conditions. Proteins were transferred on to polyvinylidene fluoride (PVDF) at 250 ma for 3 hours at 4C. Membranes were blocked with 5% milk in 0.1% triton tris buffered saline (TBS). Primary anti-TSP and secondary anti-rabbit HRP antibodies were utilized at 1∶1000 dilutions. Blots were developed using the ECL Plus system (Fisher). Purified TSP 1 and HRGP were used as controls. In some studies LL2 and B16 tumor tissues were obtained from mice at sacrifice and whole cell lysates prepared using RIPA lysis buffer. These were analyzed by western blot as above.

### Knockdown of TSP Expression by shRNA

shRNA knockdown was performed using lentiviral vectors as previously described [23], in LL2 cells. Lentiviral shRNA expressing clones SC37032-SH and SC36666-SH, targeting TSP were obtained from Santa Cruz Biotechnology. Cloned cell lines designated K1 and K2 expressing ∼60% and 25% levels of TSP1 respectively compared to cells transfected with a nontargeting control (NT) sequence. Knockdown efficiency was evaluated by RT-PCR as previously described [24], [25].

### Syngeneic Tumor Implantation Studies

C57Bl/6, *cd36* null or *hrg* null mice were anesthetized with ketamine and xylazine (IP, 50 mg/kg ketamine, 5 mg/kg xylazine). Lewis Lung Carcinoma (LL2), B16F1 Melanoma, TSR-transfected B16F1 melanoma cells, or TSP1 knockdown LL2 cells were injected subcutaneously onto the backs of eight week old male animals at a concentration of 50,000 cells/50 µl. Tumor volumes were assessed over 15–18 days using a standard formula (V = L×W^2^×0.52), which assumes a hemi elliptical shape. Mice were anesthetized at each time point. Following terminal measurement, mice were euthanized by CO_2_ and perfused with heparin (10 U/ml) and 4% paraformaldehyde. Tumors were then resected, incubated overnight in 15% sucrose and embedded in OCT. Samples were sectioned at a thickness of 10 µm. Overall cellularity and structure were evaluated by hematoxylin and eosin (H&E) staining. Blood vessel density was assessed by immunofluorescent staining using anti-VEGFR2 or VE-Cadherin antibodies. Average vessel count/mm^2^ was calculated from 6 fields of view per tumor taken at 200× magnification using a Leica DM5500B automated upright microscope system.

### TSP-2 and BAI Analysis

RNA from LL2 and B16 tumor tissue was collected using Qiagen Rneasy. Reverse-transcription PCR (RT-PCR) analysis was done as previously described [24]. Primers for actin, brain angiogenesis inhibitor (BAI) (intact vasculostatin) and thrombospondin 2 have previously been described, [25]. Threshold Cycles were normalized to actin and in the case of TSP knockdown to nontargeted values.

### Statistics

Power calculations were performed *priori* to determine group size using a standard formula, n = 2[(u_a_+u_b_)s/d]^2^, assuming variance of 20%, confidence of 95%, beta error of 0.1 and standard error of 10%. Optimal group size was calculated to be 7 individuals. Differences between groups were calculated by Student’s unpaired T-test. Outlying values were excluded using Grubb’s outlier test.

## Results

### Syngeneic Lewis Lung Tumors in *cd36* Null Mice were Larger and More Vascular than in Wildtype

LL2 cells when injected into mice lacking *cd36* produced tumors of greater size than those injected into age and sex matched wildtype mice (Figure 1A). These differences were statistically significant (P<0.05) at all time points at which tumor volumes were measurable. Mean tumor volumes in *cd36* null vs wildtype mice were 21.0 mm^3^ vs 15.8 mm^3^ at day 7, 100.4 mm^3^ vs 52.1 mm^3^ at day 10, 213.6 mm^3^ vs 136.3 mm^3^ at day 14 and 316.2 mm^3^ vs 237.7 mm^3^ at day 17 respectively. Tumors formed in *cd36* null animals displayed increased areas of necrosis as evidenced by H&E staining (data not shown) and greater vascularization (Figure 2). On average *cd36* null tumors contained 17.0 vessels/mm^2^ vs 12.2 vessels/mm^2^ in wildtype (P<0.05). These data are consistent with our hypothesis that CD36 mediates an anti-angiogenic phenotype resulting in decreased tumor vascularization and growth.

### Syngeneic Lewis Lung Tumors in *hrg* Null Mice were Smaller and Less Vascular than in Wildtype

When injected into mice lacking *hrg*, LL/2 tumors were smaller and less vascular compared to those in wildtype mice (Figure 3A). Average tumor volume in *hrg* null vs wildtype individuals were 10.4 mm^3^ vs 20.0 mm^3^ at day 7, 33.9 mm^3^ vs 49.1 mm^3^ at day 10, 93.9 mm^3^ vs 126.7 mm^3^ at day 13 and 189.6 mm^3^ vs 316.1 mm^3^ at day 17 respectively. Differences at all points day 7 and beyond were significant at P<0.05. Tumors in *hrg* null mice displayed less necrosis (data not shown) and were characterized by decreased vasculature compared with wildtype (Figure 4); on average *hrg* null tumors contained 7.1 vessels/mm^2^ vs 13.6 vessels/mm^2^ in wildtype (P<0.05). These data are consistent with our hypothesis that HRG modulates CD36-TSR anti-angiogenic signaling.

### Tumor Cell TSR Expression is Required for Regulation of Syngeneic Tumor Growth and Vascularity by Genetic Manipulation of *cd36* or *hrg*

In sharp contrast to the results seen with LL2 cells, implantation of B16F1 melanoma cells resulted in tumors of similar size (Figure 1B and 3B) and vascularity (not shown) regardless of genetic background of the host. We hypothesized that these differences may relate to differing levels of TSR protein expression and indeed immunoblot analysis of conditioned media from the tumor cell lines showed readily detectable TSP-1 in the postculture media from LL2 cells, but not from B16F1 cells or tumor tissue (Figures 5A and 5B). RT-PCR confirmed these results, with elevated expression of TSP-1 in LL2 tumor tissue (Figure 5C).

We therefore generated stably transfected B16F1 cell lines that expressed and secreted recombinant TSP-1 TSR domains. As shown in Figure 5B, Clone 11 expressed abundant TSR and was used for all further experiments. TSR transfection restored responsiveness to the CD36/HRGP system. TSR expressing B16F1 cells produced larger and more vascular tumors in *cd36* null mice (Figures 6A, 7A left and 7B top) and smaller and less vascular tumors in *hrgp* null mice (Figures 6B, 7A right and 7B bottom) when compared to age and sex matched wildtype controls. Average tumor volume in *cd36* null vs wildtype individuals were 27.8 mm^3^ vs 17.5 mm^3^ at day 8 (P = 0.08); 67.7 mm^3^ vs 37.8 mm^3^ at day 11 (P<0.05); 170.5 mm^3^ vs 98.0 mm^3^ at day 14 (P<0.05); and 685.1 mm^3^ vs 394.7 mm^3^ at day 18 (p = 0.06). On average *cd36* null tumors contained 16.3 vessels/mm^2^ vs 9.1 vessels/mm^2^ in wildtype (P<0.05). In the *hrgp* null mice the average tumor volumes compared to wildtype were 5.8 mm^3^ vs 53.2 mm^3^ at day 11; 87.9 mm^3^ vs 255.3 mm^3^ at day 15; and 211.0 mm^3^ vs 651.7 mm^3^ at day 18. All of these differences were significant at P<0.05. The tumors formed in *hrg* null animals were more vascularized with on average 5.4 vessels/mm^2^ vs 10.1 vessels/mm^2^ in wildtype (P<0.05). These data further support our hypothesis that CD36-TSR interaction mediates an anti-angiogenic phenotype with modulation by HRG.

To complement the B16F1 TSP1 overexpression studies we also examined the effect of LL2 cell TSP1 knockdown on tumor volume and vascularity. Knockdown of TSP1 was confirmed by quantitative RT-PCR (Figure 5E). As before the control NT LL2 cells formed larger tumors in cd36 null mice as compared to wildtype (not shown). In the wildtype mouse recipients TSP1 knockdown resulted in larger tumors than seen with the NT control cells. The effects were statistically significant for both K1 and K2 cells at days 7 and 15 (Figure 6C) and the degree of difference was proportional to the level of knockdown. No differences among the NT, K1 and K2 groups were observed in cd36 null mice (Figure 6D). Vessel density was also significantly decreased in the TSP1 knockdown tumors (8.2 vessels/mm^2^ in the control group compared with 15.9 vessels/mm^2^ in the knockdown tumors (Figure 7C). Taken together these data further support the claim that TSP expression mediates the action of CD36 and HRG.

It should be noted that other TSR containing proteins, such as TSP2 and BAI (Figures S1A and S1B respectively) were detected in B16F1 and LL2 tumor tissue by RT-PCR. This presents the opportunity for multiple levels of angiogenic modulation with additional study required to further characterize the interplay between TSR containing proteins.

## Discussion

CD36-TSR signaling inhibits microvascular endothelial cell migration, proliferation and tube formation in *in vitro* and *in* *vivo* models. Our group has shown that this important endogenous anti-angiogenic system can be dampened by HRG, a protein with structural homology to CD36 that acts as a decoy for TSR. *In vitro* assays of microvascular endothelial cell migration, proliferation, and tube formation; and *in* *vivo* assays of angiogenesis in mouse corneal micropockets and implanted matrigel showed that addition of exogenous HRG blocks TSP-1, TSP-2 and vasculostatin binding to CD36 and thereby inhibits TSR-mediated vascular cell responses [9], [10], [26]. HRG circulates in high concentrations, can be released from activated platelets, and accumulates in perivascular matrix; thus it is “poised” to serve an important role in regulating microvascular CD36-TSR signaling *in vivo* [27], [28]. This may have particular relevance to tumor angiogenesis since HRG has been shown localize in the stromal connective tissue of human tumors, including breast cancer and glioblastoma, and to mask the TSR domain of TSP [10], [26], [29]. The potential importance of this system in carcinogenesis is supported by abundant data showing that *TSP1* has potent tumor suppressor activity and that genetic or epigenetic down-regulation of TSP-1 expression is associated with progression of numerous human cancers and enhanced tumor angiogenesis. We thus hypothesized that accumulation of HRG in the tumor microenvironment would promote tumor growth, similar to loss of tumor cell TSR expression, and that down-regulation of the receptor, CD36 would have an opposite effect.

In the experiments described in this manuscript we used mouse genetic models to provide direct evidence in support of this hypothesis. In the absence of CD36, transplanted syngeneic tumors were larger and displayed increased vascularity, while in the absence of HRG, tumors were smaller and displayed less vascularity. Importantly, these host-mediated effects required production and secretion of TSR-containing protein by the transplanted tumor. TSP-1 secreting LL2 cells were sensitive to loss of CD36 or HRG, while TSP-1 negative B16F1 melanoma cells were not sensitive unless they were stably transfected with a TSR-expressing plasmid. Furthermore, knockdown of TSP-1 in the LL2 cells resulted in loss of CD36 sensitivity. Our data suggest that tumor cells could induce a state of functional TSR deficiency and hence promote angiogenesis and tumor growth) by remodeling their micro-environment to down-regulate microvascular CD36 expression and/or up-regulate accumulation of HRGP. In regard to the former, we recently found that lysophosphatidic acid (LPA) activates a protein kinase D-mediated signaling pathway in microvascular endothelial cells that transcriptionally silences *cd36* and thereby promotes angiogenesis [30]. Since both tumor cells and inflammatory cells are potential sources of LPA, this could be highly relevant to tumor biology.

HRG accumulation in tumor microenvironments would be expected to relate to at least two processes known to promote tumor growth and metastasis – VEGF expression and platelet activation [31], [32]. In addition to promoting angiogenesis, VEGF is a potent microvascular permeability factor that contributes to the “leaky” vasculature of tumor beds [33]. In this milieu, plasma proteins such as HRG escape from the confines of the vessel and permeate into the tumor bed. Similarly, platelet-tumor cell interactions have been studied for many years and are known to promote both tumor growth and thrombosis [34]. Platelet accumulation and activation in a tumor microenvironment would have many effects, including release of both TSP-1 and HRG.

HRG was first characterized in 1978 as a molecule which bound heme and certain metal ions [35]. Today, it is viewed as an adapter protein due to its multi-domain nature and multiple ligand binding capacity, and has been implicated in diverse functions including immunity, thrombosis, cell adhesion and angiogenesis [36]–[38]. The potential to develop HRG as a novel therapeutic target to regulate angiogenesis is complicated by reports from other groups showing in contrast to our work, that HRG has anti-angiogenic activity [39], [40]. The mechanism for this activity has not been defined, but it is mediated by the histidine-proline rich region of the protein. Our genetic models and abundant *in vitro* and *in vivo* studies using intact, native HRG strongly support a pro-angiogenic role for HRG in the presence of TSR proteins and did not show any anti-angiogenic activity, even in the absence of TSR proteins. The most likely explanation for this apparent controversy is that the anti-angiogenic activity requires proteolytic release of the histidine/proline-rich domain. Whether there is an endogenous pathway to release the domain has not been demonstrated, but precedent exists for proteolytic peptide fragments having opposite biological activity than their “parent” protein [41]. In addition, multiple groups have reported tumor inhibitory properties of intact HRG [29], [42]. These studies performed using hepatocellular carcinoma, fibrosarcoma and pancreatic carcinomas have highlighted the modulation of tumor associated macrophages by HRG. In these studies, HRG shifts infiltrating macrophages toward a pro-angiogenic phenotype also characterized by inhibition of immune cells such as dendritic and cytotoxic T cells. Thus, HRG appears to possess multiple mechanisms by which it may modulate angiogenesis and tumor growth. Additional work is required to elucidate the possible role tissue associated macrophages may play in CD36-HRG-TSP signaling as well as the anti-tumor properties of HRG in LL2 and B16F1 tumors. It is unclear why HRG appears to possess pro and anti-angiogenic properties in varying tumor types.

In summary, we showed in these studies that modulating tumor cell expression of TSR proteins or expression in the non-transformed tumor microenvironment of CD36 or HRG had significant impact on tumor angiogenesis and tumor growth. Numerous pro- and anti-angiogenic therapies are in clinical trials, among them ABT-510 and ABT-898, which are peptide mimetics of the TSR domain of TSP-1. These compounds have shown potential for treatment of cancer suggesting that targeting CD36 or HRG could present effective alternative approaches to enhance or inhibit TSR action [43]–[45].

## Supporting Information

Figure S1 **Thrombospondin 2 (TSP2) and Brain angiogenesis inhibitor (BAI) mRNA expression is detected in LL2 and B16F1 tumor tissue.** B16F1 and LL2 tumor tissue was analyzed by RT-PCR for expression of TSP2 (**A**) and BAI (**B**). Detectable levels of TSP2 and BAI were observed in both tumor types with inhanced expression in LL2 vs B16F1 tumors.
(TIF)

## References

[1] Folkman J (2006). Angiogenesis.. Annu Rev Med.

[2] Kerbel RS (2008). Tumor angiogenesis.. N Engl J Med.

[3] Nussenbaum F, Herman IM (2010). Tumor angiogenesis: insights and innovations.. J Oncol,132641.

[4] Dawson D, Pearce S, Zhong R, Silverstein R, Frazier W (1997). CD36 mediates the In vitro inhibitory effects of thrombospondin-1 on endothelial cells.. J Cell Biol.

[5] Kaur B, Cork S, Sandberg E, Devi N, Zhang Z (2009). Vasculostatin inhibits intracranial glioma growth and negatively regulates in vivo angiogenesis through a CD36-dependent mechanism.. Cancer Res.

[6] Nicholson A, Frieda S, Pearce A, Silverstein R (1995). Oxidized LDL binds to CD36 on human monocyte-derived macrophages and transfected cell lines. Evidence implicating the lipid moiety of the lipoprotein as the binding site.. Arterioscler Thromb Vasc Biol.

[7] Coburn C, Knapp F, Febbraio M, Beets A, Silverstein R (2000). Defective uptake and utilization of long chain fatty acids in muscle and adipose tissues of CD36 knockout mice.. J Biol Chem.

[8] Pearce S, Wu J, Silverstein R (1995). Recombinant GST/CD36 fusion proteins define a thrombospondin binding domain. Evidence for a single calcium-dependent binding site on CD36.. J Biol Chem.

[9] Simantov R, Febbraio M, Silverstein R (2005). The antiangiogenic effect of thrombospondin-2 is mediated by CD36 and modulated by histidine-rich glycoprotein.. Matrix Biol.

[10] Klenotic P, Huang P, Palomo J, Kaur B, Van Meir E (2010). Histidine-rich glycoprotein modulates the anti-angiogenic effects of vasculostatin.. Am J Pathol.

[11] Jimenez B, Volpert O, Crawford S, Febbraio M, Silverstein R (2000). Signals leading to apoptosis-dependent inhibition of neovascularization by thrombospondin-1.. Nat Med.

[12] Rege T, Stewart J, Dranka B, Benveniste E, Silverstein R (2009). Thrombospondin-1-induced apoptosis of brain microvascular endothelial cells can be mediated by TNF-R1.. J Cell Physiol.

[13] Volpert O, Zaichuk T, Zhou W, Reiher F, Ferguson T (2002). Inducer-stimulated Fas targets activated endothelium for destruction by anti-angiogenic thrombospondin-1 and pigment epithelium-derived factor.. Nat Med.

[14] Gutierrez L, Suckow M, Lawler J, Ploplis V, Castellino F (2003). Thrombospondin 1–a regulator of adenoma growth and carcinoma progression in the APC(Min/+) mouse model.. Carcinogenesis.

[15] Streit M, Riccardi L, Velasco P, Brown L, Hawighorst T (1999). Thrombospondin-2: a potent endogenous inhibitor of tumor growth and angiogenesis.. Proc Natl Acad Sci U S A.

[16] Kaur B, Brat D, Devi N, Van Meir E (2005). Vasculostatin, a proteolytic fragment of brain angiogenesis inhibitor 1, is an antiangiogenic and antitumorigenic factor.. Oncogene.

[17] Yang Q, Liu S, Tian Y, Salwen H, Chlenski A (2003). Methylation-associated silencing of the thrombospondin-1 gene in human neuroblastoma.. Cancer Res.

[18] Crombie R, Silverstein R, MacLow C, Pearce S, Nachman R (1998). Identification of a CD36-related thrombospondin 1-binding domain in HIV-1 envelope glycoprotein gp120: relationship to HIV-1-specific inhibitory factors in human saliva.. J Exp Med.

[19] Jones A, Hulett M, Parish C (2005). Histidine-rich glycoprotein: A novel adaptor protein in plasma that modulates the immune, vascular and coagulation systems.. Immunol Cell Biol.

[20] Hulett M, Parish C (2000). Murine histidine-rich glycoprotein: cloning, characterization and cellular origin.. Immunol Cell Biol.

[21] Febbraio M, Abumrad N, Hajjar D, Sharma K, Cheng W (1999). A null mutation in murine CD36 reveals an important role in fatty acid and lipoprotein metabolism.. J Biol Chem.

[22] Tsuchida-Straeten N, Ensslen S, Schäfer C, Wöltje M, Denecke B (2005). Enhanced blood coagulation and fibrinolysis in mice lacking histidine-rich glycoprotein (HRG).. J Thromb Haemost.

[23] Li Z, Bao S, Wu Q, Wang H, Eyler C (2009). Hypoxia-inducible factors regulate tumorigenic capacity of glioma stem cells.. Cancer Cell.

[24] Lathia JD, Gallagher J, Heddleston JM, Wang J, Eyler CE (2010). Integrin alpha 6 regulates glioblastoma stem cells.. Cell Stem Cell.

[25] Kodama J, Hashimoto I, Seki N, Hongo A, Yoshinouchi M (2001). Thrombospondin-1 and -2 messenger RNA expression in invasive cervical cancer: correlation with angiogenesis and prognosis.. Clin Cancer Res 2001.

[26] Simantov R, Febbraio M, Crombie R, Asch A, Nachman R (2001). Histidine-rich glycoprotein inhibits the antiangiogenic effect of thrombospondin-1.. J Clin Invest.

[27] Leung L, Harpel P, Nachman R, Rabellino E (1983). Histidine-rich glycoprotein is present in human platelets and is released following thrombin stimulation.. Blood.

[28] Leung L (1993). Histidine-rich glycoprotein: an abundant plasma protein in search of a function.. J Lab Clin Med.

[29] Rolny C, Mazzone M, Tugues S, Laoui D, Johansson I (2011). HRG inhibits tumor growth and metastasis by inducing macrophage polarization and vessel normalization through downregulation of PlGF.. Cancer Cell.

[30] Ren B, Hale J, Srikanthan S, Silverstein R (2011). Lysophosphatidic acid suppresses endothelial cell CD36 expression and promotes angiogenesis via a PKD-1-dependent signaling pathway.. Blood.

[31] Palumbo J, Talmage K, Massari J, La Jeunesse C, Flick M (2005). Platelets and fibrin(ogen) increase metastatic potential by impeding natural killer cell-mediated elimination of tumor cells.. Blood.

[32] Rowe D, Huang J, Kayton M, Thompson R, Troxel A (2000). Anti-VEGF antibody suppresses primary tumor growth and metastasis in an experimental model of Wilms’ tumor.. J Pediatr Surg 35: 30–32; discussion 32–33.

[33] Karathanasis E, Chan L, Karumbaiah L, McNeeley K, D’Orsi C (2009). Tumor vascular permeability to a nanoprobe correlates to tumor-specific expression levels of angiogenic markers.. PLoS One.

[34] Nijziel M, van Oerle, Hillen H, Hamulyak K (2006). From Trousseau to angiogenesis: the link between the haemostatic system and cancer.. Neth J Med.

[35] Morgan W (1978). Human serum histidine-rich glycoprotein. I. Interactions with heme, metal ions and organic ligands.. Biochim Biophys Acta.

[36] Shatsky M, Saigo K, Burdach S, Leung L, Levitt L (1989). Histidine-rich glycoprotein blocks T cell rosette formation and modulates both T cell activation and immunoregulation.. J Biol Chem.

[37] Leung L (1986). Interaction of histidine-rich glycoprotein with fibrinogen and fibrin.. J Clin Invest.

[38] Chang N, Leu R, Rummage J, Anderson J, Mole J (1992). Regulation of macrophage Fc receptor expression and phagocytosis by histidine-rich glycoprotein.. Immunology.

[39] Juarez J, Guan X, Shipulina N, Plunkett M, Parry G (2002). Histidine-proline-rich glycoprotein has potent antiangiogenic activity mediated through the histidine-proline-rich domain.. Cancer Res.

[40] Dixelius J, Olsson A, Thulin A, Lee C, Johansson I (2006). Minimal active domain and mechanism of action of the angiogenesis inhibitor histidine-rich glycoprotein.. Cancer Res.

[41] Struman I, Bentzien F, Lee H, Mainfroid V, D’Angelo G (1999). Opposing actions of intact and N-terminal fragments of the human prolactin/growth hormone family members on angiogenesis: an efficient mechanism for the regulation of angiogenesis.. Proc Natl Acad Sci U S A.

[42] Tugues S, Honjo S, Konig C, Noguer O, Hedlund M (2012). Genetic Deficiency in Plasma Protein HRG Enhances Tumor Growth and Metastasis by Exacerbating Immune Escape and Vessel Abnormalization.. Cancer Res.

[43] Hoekstra R, de Vos F, Eskens F, Gietema J, van der Gaast A (2005). Phase I safety, pharmacokinetic, and pharmacodynamic study of the thrombospondin-1-mimetic angiogenesis inhibitor ABT-510 in patients with advanced cancer.. J Clin Oncol.

[44] Markovic S, Suman V, Rao R, Ingle J, Kaur J (2007). A phase II study of ABT-510 (thrombospondin-1 analog) for the treatment of metastatic melanoma.. Am J Clin Oncol.

[45] Garside S, Henkin J, Morris K, Norvell S, Thomas F (2010). A thrombospondin-mimetic peptide, ABT-898, suppresses angiogenesis and promotes follicular atresia in pre- and early-antral follicles in vivo.. Endocrinology.

