# Supplementary figures and images for: Context Dependent Role of the CD36 - Thrombospondin - Histidine-Rich Glycoprotein Axis in Tumor Angiogenesis and Growth
Source: PLoS One. 2012 Jul 10;7(7):e40033. doi: 10.1371/journal.pone.0040033 (PMC3393734; doi:10.1371/journal.pone.0040033)

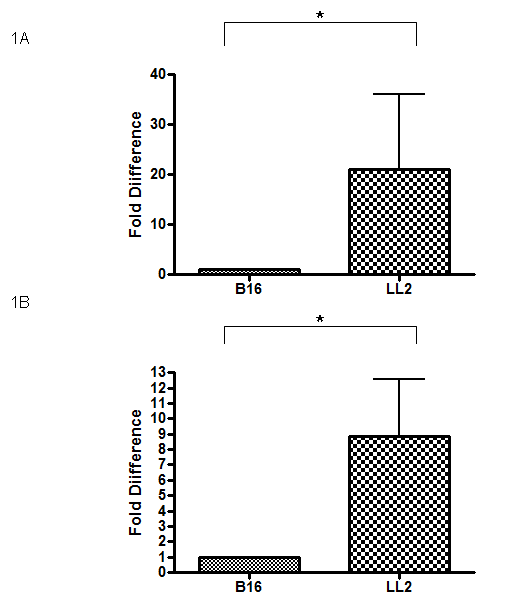

Supplement: Figure S1 — Thrombospondin 2 (TSP2) and Brain angiogenesis inhibitor (BAI) mRNA expression is detected in LL2 and B16F1 tumor tissue. B16F1 and LL2 tumor tissue was analyzed by RT-PCR for expression of TSP2 (A) and BAI (B). Detectable levels of TSP2 and BAI were observed in both tumor types with inhanced expression in LL2 vs B16F1 tumors. (TIF) [file pone.0040033.s001.tif]
